# Editorial: ‘To err is human, it takes a computer to really foul things up!’*

**DOI:** 10.1155/S1463924685000360

**Published:** 1985

**Authors:** Kent K. Stewart

**Affiliations:** Virginia Polytechnic Institute and State University State University Blacksburg Virginia 24061 USA


Journal of Automatic Chemistry/*Journal of Clinical Laboratory Automation*, Vol. 7, No. 4 (October–December 1985), p. 169

### Editorial: 'To err is human, it takes a computer to really foul things up!'*

The increasing use of computers and other automation tools in chemical assays has the potential for significantly improving the productivity of our laboratories. However, unless these assay systems are carefully monitored, laboratory automation can become a nightmare. The data produced by modern computerized automated instruments are often believed simply because they are produced by modern instruments, particularly if the instruments have computerized output. Some of these data are incorrect. The classic computer concept of GIGO (Garbage In, Garbage Out) holds true in automated assays. Accurate data are not commonly obtained by the automation of a poor method. There are other considerations which are more subtle and possibly more serious.

While automation and computerization reduce the necessity of human intervention and thus human error, the inherent quality assurance provided by the human analyst is lost. With many modern instruments and laboratory data systems, the analysts no longer control the instrumentation, and/or the calculation of the final results. Often the algorithms for the assay and computational operations have been designed by engineers and not by analytical chemists. Sometimes these algorithms are considered proprietary, and the user of the system is not permitted to know the details of processes of the assay or the computations. Such events should make even strong-hearted analytical chemists quake with fear or anger or both. A new trend is to also package the assay chemistry. Thus there is a potential for completely packaged black-box assays. Such systems can be very useful; they also can be a modern form of magic.

A properly designed automated assay system should provide documented instrument set-up and control, documented computation algorithms, files of all key parameters, and other built in quality-control procedures necessary for individually validated data sets. It is incumbent upon the analytical chemists designing and building such systems to ensure that these features are incorporated into the new instrumentation and assay systems. Furthermore, all aspects of the assay system and instrumentation must be completely documented.

Unfortunately, it is unlikely that instrument companies will incorporate such quality-control features without prodding. Analysts will have to communicate these requirements to the instrument companies and probably will eventually have to insist that these quality-control components be a part of all newly purchased instrumentation and assay systems.

In addition, analysts using automated assay systems must establish the appropriate quality-control procedures for their automated assays. Hopefully these procedures will include the use of the appropriate control and pool samples to validate individual assay results. All of the above features must be incorporated into modern automated assay procedures. Any less carries the potential for disaster.

**Kent K. Stewart**
Editor: USA
*Virginia Polytechnic Institute and State University,*
*State University, Blacksburg, Virginia*
*24061, USA*

* Sign in a computer center.

